# Pharmacokinetics and safety of brivaracetam in neonates with repeated electroencephalographic seizures: A multicenter, open‐label, single‐arm study

**DOI:** 10.1002/epi4.12875

**Published:** 2024-01-11

**Authors:** Ronit Pressler, Geraldine Boylan, Eugene Dempsey, Kerstin Alexandra Klotz, Walter Krauwinkel, Edgar Will, Diego Morita, Florin Floricel, Jan‐Peer Elshoff, John van den Anker

**Affiliations:** ^1^ Department of Clinical Neurophysiology, Great Ormond Street Hospital and Clinical Neuroscience UCL‐GOS Institute of Child Health London UK; ^2^ INFANT Research Centre and Department of Paediatrics and Child Health Cork Ireland; ^3^ Department of Neuropediatrics and Muscle Disorders, Medical Center University of Freiburg Freiburg Germany; ^4^ UCB Pharma Braine‐l'Alleud Belgium; ^5^ UCB Pharma Monheim am Rhein Germany; ^6^ UCB Pharma Morrisville North Carolina USA; ^7^ Children's National Hospital Washington District of Columbia USA

**Keywords:** antiseizure medication, neonatal seizures, PETITE study, tolerability, video‐electroencephalography

## Abstract

**Objective:**

To evaluate the pharmacokinetics (PK), safety, and tolerability of brivaracetam (BRV) in neonates with repeated electroencephalographic seizures not controlled with previous antiseizure medications (ASMs).

**Methods:**

Phase 2/3, multicenter, open‐label, single‐arm study (N01349/NCT03325439) in neonates with repeated electroencephalographic seizures (lasting ≥10 s) confirmed by video‐electroencephalography, and inadequate seizure control with at least one ASM. A screening period (up to 36 h) was followed by a 48‐h evaluation period during which patients received 0.5 mg/kg BRV twice daily (b.i.d) intravenously (IV). Patients who benefitted from BRV (investigator's opinion) could continue 0.5 mg/kg b.i.d (IV or oral solution) in an extension period. Outcomes included plasma concentrations of BRV following the first dose (primary), and incidence of treatment‐emergent adverse events (TEAEs).

**Results:**

Six patients (median [range] postnatal age: 1.5 [1.0, 6.0] days) received ≥1 dose of BRV. All six patients completed the evaluation period; two entered and completed the extension period. Overall (evaluation and extension periods), three patients received one dose of 0.5 mg/kg BRV and three received more than one dose. The median (range) duration of exposure to BRV (IV and oral solution) was 1.5 (1.0, 29.0) days (*n* = 6). At 0.5–1, 2–4, and 8–12 h following IV BRV administration, the GeoMean (GeoCV) plasma concentrations of BRV were 0.53 mg/L (15.40% [*n* = 5]), 0.50 mg/L (28.20% [*n* = 6]), and 0.34 mg/L (13.20% [*n* = 5]), respectively. Individual and population BRV PK profiles were estimated, and individual PK parameters were calculated using Bayesian feedback. The observed concentrations were consistent with the predicted PK. Three patients experienced four TEAEs, none of which were considered related to BRV.

**Significance:**

BRV plasma concentrations in neonates were consistent with data in older children receiving BRV oral solution, and with data from adults receiving a nominal IV dose of 25 mg b.i.d. BRV was well tolerated, with no drug‐related TEAEs reported.

**Plain Language Summary:**

Few drugs are available to treat seizures in newborn babies. Brivaracetam is approved to treat focal‐onset seizures in children and adults in Europe (patients 2 years of age and older) and the United States (patients 1 month of age or older). In this study, six newborns with repeated seizures were treated with intravenous brivaracetam. The study doctors took samples of blood from the newborns and measured the levels of brivaracetam. The concentrations of brivaracetam in the newborns’ blood plasma were consistent with data from studies in older children and in adults. No brivaracetam‐related medical problems were reported.

**Key points**

- Six neonates (median postnatal age: 1.5 days) with electroencephalographic seizures received at least one dose of 0.5 mg/kg b.i.d IV BRV.
- Plasma concentrations of BRV were evaluated following the first dose on Day 1, and another dose on Day 2.
- BRV plasma concentration in neonates was consistent with PK observed in older children, and in adults on a nominal IV dose of 25 mg b.i.d.
- BRV was well tolerated, and no drug‐related treatment‐emergent adverse events were reported.

## 1 INTRODUCTION

Seizures during the neonatal period are the most common neurological emergency.^1^ Neonatal seizures occur in 1 to 4 per 1000 live births, and are seen much more frequently in preterm neonates (11.1 per 1000 live births).^2^, ^3^ Seizures in neonates differ from those of older children and adults^1^; most seizures in the neonatal period are acute symptomatic (or acute provoked, reactive) and many are electrographic‐only. Neonatal seizures are most commonly caused by hypoxic‐ischemic encephalopathy (HIE), intracranial hemorrhage, cerebral infarction, or infection.^4^, ^5^ Seizures in neonates are associated with adverse neurodevelopmental outcomes.^6^

Few treatment options are available for the management of neonatal seizures. The Neonatal Task Force of the International League Against Epilepsy (ILAE) recently published evidence‐based recommendations regarding antiseizure medication (ASM) management in neonates.^7^ Phenobarbital (PB) is recommended as the first‐line treatment for neonatal seizures, unless channelopathy is the likely etiology (based on family history), in which case phenytoin (PHT) or carbamazepine should be used. PHT, levetiracetam (LEV), midazolam (MDZ), or lidocaine (LDC) are recommended as second‐line ASMs for non‐responders.

With the exception of PB (which was not approved at the time of the study),^8^, ^9^ ASMs are used off‐label. As such, there is an urgent need to develop additional ASMs for the treatment of neonatal seizures. Brivaracetam (BRV) is currently indicated for adjunctive treatment of focal (partial‐onset) seizures in patients 2 years of age and older in the European Union,^10^ and as monotherapy and adjunctive treatment in patients 1 month of age and older in the United States.^11^ The ILAE guidelines note that, although BRV is an emerging ASM for neonatal seizures, its use could not be recommended due to the lack of published controlled studies on its efficacy or safety in neonates.^7^

The objective of this open‐label study was to evaluate the pharmacokinetics (PK), safety, and tolerability of BRV in neonates with repeated electroencephalographic seizures not controlled with previous ASMs, and to identify the optimal BRV dose for the treatment of patients planned to be enrolled for evaluation of efficacy.

## 2 METHODS

### 2.1 Study design

N01349 (the PETITE study/ClinicalTrials.gov: NCT03325439) was a phase 2/3, multicenter, open‐label, single‐arm, two‐step study. We report data from Step One of the study (Exploratory Cohort), which aimed to identify the optimal BRV dose (confirm or adapt the dose predictions of the initial modeling) for the treatment of patients in Step Two (Confirmatory Cohort). Step Two was planned to evaluate the efficacy of BRV; it was initiated in November 2020 but was stopped 13 months later (following agreement with the European Medicines Agency Paediatric Committee) due to the lack of enrollment of eligible study patients.

The study was conducted in accordance with Good Clinical Practice, the Declaration of Helsinki, and local laws. The trial protocol and amendments were approved by Independent Ethics Committees, as defined in local regulations. Parents or legal representatives of patients provided written informed consent before study participation. During the study, continuous consent was utilized via meetings between the investigator and the parent or legal representatives of the patient to discuss the patient's ongoing care and study participation. This enhanced overall comprehension of the study and associated potential risks.

### 2.2 Patient population

Eligible patients were male or female neonates (≥34 weeks of corrected gestational age [CGA], term neonates up to 27 days of postnatal age [PNA], and preterm neonates up to 40 weeks of CGA and 27 days of PNA) with bodyweight ≥2.3 kg at enrollment; with repeated electroencephalographic neonatal seizures (defined as a seizure lasting ≥10 s^12^) assessed by continuous video‐electroencephalography (EEG), and without adequate seizure control before or at the time of enrollment after treatment with at least one of the following ASMs: LEV, LDC, MDZ, PB, or PHT (first‐line, second‐line, or subsequent treatment, which may have started before admission to the study site; choice of treatment, dose, and dosing regimen at the investigator's discretion). Patients must have had confirmation on video‐EEG ≥2 min of cumulative electroencephalographic seizures or at least three identifiable electroencephalographic seizures within up to 1 h before entering the evaluation period. Patients were permitted concomitant hypothermia treatment.

The main exclusion criteria included seizures responding to previous ASM treatment immediately before BRV treatment, pyridoxine treatment, or correction of metabolic disturbances; extra corporeal membrane oxygenation; seizures related to prenatal maternal drug use or drug withdrawal; known severe disturbance of hemostasis, or a poor prognosis for survival, as judged by the investigator. Patients who had twice the upper limit of normal of any of the following were excluded: aspartate aminotransferase, alanine aminotransferase, and alkaline phosphatase. For patients with perinatal asphyxia, elevation of aspartate aminotransferase, alanine aminotransferase, or alkaline phosphatase <5 times the upper limit of normal was acceptable provided that the initial and peak elevation of liver function tests occurred within 5 days after birth, and the time course of liver function test elevation was compatible with hepatic injury due to perinatal asphyxia. The determination of upper limit of normal was based on the study patient's gestational age (GA) and the site's normal range values for the respective GA. Patients were excluded if they had direct (conjugated) bilirubin levels >2 mg/dL, required or were expected to require phototherapy or exchange transfusion due to elevated bilirubin, or had rapidly increasing bilirubin that may have precluded study participation (discretion of the investigator). Patients with severe cardiac conditions, including patients with a diagnosis or signs of long QT syndrome; with a family history of long QT syndrome (patients enrolled in Germany only); and with rhesus incompatibility, with or without Rh‐isoimmunization (patients enrolled in the Czech Republic only) were also excluded.

### 2.3 Treatment schedule

**FIGURE 1:**
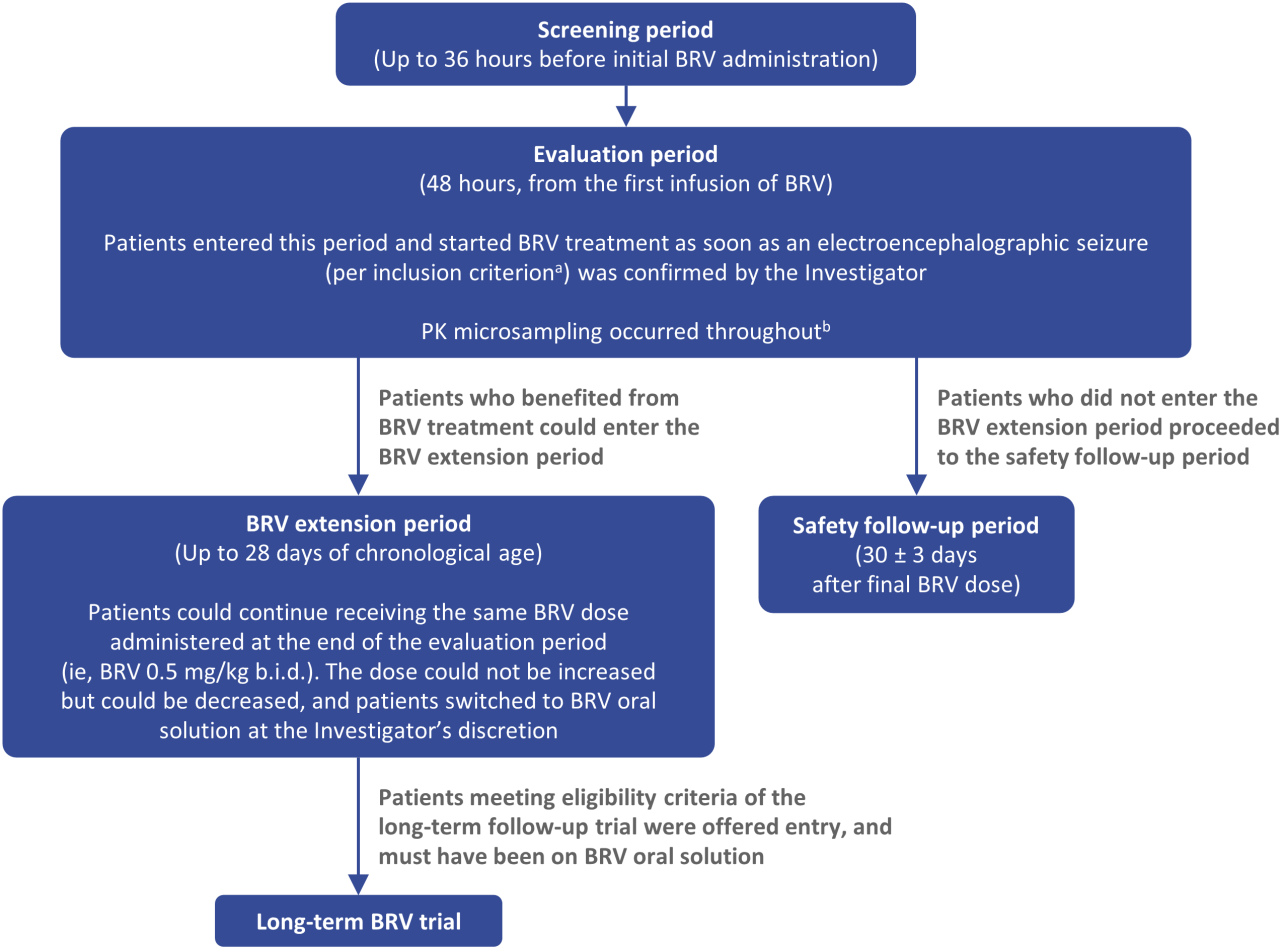
Study design. ^a^Confirmation on video‐EEG of ≥2 min of cumulative electroencephalographic seizures or at least three identifiable electroencephalographic seizures before entering the evaluation period (an electroencephalographic seizure was defined as a seizure lasting ≥10 s on video‐EEG), despite receiving previous ASM for the treatment of electroencephalographic seizures. The occurrence of electroencephalographic seizures during an up to 1‐h period had to be confirmed either by the local or central video‐EEG reader before drug administration; ^b^Following the first BRV infusion, three to six blood microsamples (60 μL/sample) were collected from each patient. On Day 1, samples were collected at 30–60 min after the start of BRV infusion and then at 2–4 and 8–12 h after the start of the most recent BRV infusion. On Day 2, samples were only collected if the patient received BRV on that day; the timepoints for sample collection were 30–60 min, 2–4 h, and 8–12 h after the start of the most recent BRV infusion. In addition, opportunistic blood samples for PK analysis may have been used at the investigator's discretion at any time during the evaluation period. ASM, antiseizure medication; b.i.d, twice daily; BRV, brivaracetam; EEG, electroencephalography; PK, pharmacokinetic.

Step One of the study (Exploratory Cohort) included a screening period (up to 36 h before first administration of BRV) and a 48‐h evaluation period, followed by entry into either a BRV extension period or safety follow‐up period. Eligible patients started BRV treatment as soon as the occurrence of an electroencephalographic seizure was confirmed by video‐EEG (which was supported by remote monitoring of EEG). Patients were dosed with 0.5 mg/kg BRV twice daily (b.i.d) as an approximate 15‐min intravenous (IV) infusion during the evaluation period according to each site's procedures. The first IV BRV infusion marked the starting point of the 48‐h evaluation period (Figure 1). At the discretion of the investigator, additional BRV doses (0.5 mg/kg b.i.d IV) could be administered every 12 h (up to a maximum of three doses during the 48‐h evaluation period). This treatment regimen of BRV in neonates was four‐fold less than the highest dose of 4 mg/kg/day (2 mg/kg b.i.d) used previously in infants ≥1 month of age.^13^ Standard of care treatment with ASMs continued in parallel with BRV treatment.

Patients who benefited from BRV treatment (in the investigator's opinion) could enter the BRV extension period (up to 28 [+7] days of PNA). These patients continued on a BRV dose of 0.5 mg/kg b.i.d, and could switch from IV administration to oral solution, at the discretion of the investigator. Patients who switched to oral BRV could enter a long‐term follow‐up study after the BRV extension period (N01266; ClinicalTrials.gov: NCT01364597). Patients who did not enter the BRV extension period proceeded to the safety follow‐up period, with a final visit 30 ± 3 days after the final BRV administration.

### 2.4 Outcomes

The primary PK variable was the plasma concentrations of BRV following the first dose. Other PK variables were plasma concentrations of BRV following dose on Day 2, plasma PK parameters of BRV after IV administration (area under the curve [AUC], maximum plasma concentration, time to reach maximum plasma concentration, elimination half‐life, volume of distribution, and clearance); plasma concentrations of BRV metabolites (acid metabolite, hydroxy metabolite, and the hydroxy acid metabolite), and plasma concentrations of concomitant PB and PHT (if administered).

The secondary safety variable was the incidence of treatment‐emergent adverse events (TEAEs), reported and assessed by the investigators. Other safety variables were change from baseline in vital signs (systolic/diastolic blood pressure [SBP/DBP], heart rate, respiration rate, oxygen saturation, and body temperature); physical and neurological examinations during the evaluation period; change from baseline in safety laboratory tests, Neonatal Pain, Agitation, and Sedation Scale (N‐PASS) score during the evaluation period; change from baseline in biometric parameters (length, body weight, head circumference) at the safety follow‐up visit, and mechanical ventilation during the evaluation period.

### 2.5 Assessments

#### 2.5.1 PK analyses

During the 48‐h evaluation period, up to six venous or arterial blood microsamples (60 μL/sample) were collected from each patient. Three BRV PK samples of blood were collected at three predetermined timepoints per day (30–60 min, 2–4 h, and 8–12 h after the start of the most recent IV BRV administration). ASM PK samples were collected 3 h after the start of the initial IV BRV administration.

For predicted BRV profiles, PK parameters were estimated using Bayesian feedback performed using the nlmixr package (version 1.1.1‐3) implemented in R software (version 3.6.2), where bioavailability was assumed to be 100%, and where infusion bypassed the oral absorption model component. Individual‐specific PK parameters were determined using the patient's dosing history and BRV concentration assessments, where prior information on the typical population parameters in children (<16 years of age) and their inter‐individual variability was taken into account. The prior information was provided by a model that describes BRV PK after oral administration in pediatric patients with epilepsy aged 1 month to 16 years, using a one‐compartment first order absorption model with allometric scaling of clearance and central volume to body weight using fixed allometric constants of 0.75 and 1, respectively.^14^

#### 2.5.2 Safety analyses

All TEAEs were recorded, and the seriousness determined; TEAEs were coded using the Medical Dictionary for Regulatory Activities (MedDRA®) Version 18.1, and followed until they were resolved, had stable sequelae, were no longer clinically significant as determined by the investigator, or the patient was lost to follow‐up. Video‐EEG monitoring performed during the evaluation period was assessed for the study patient's reactivity, in addition to the interpretation of the N‐PASS in terms of sedation status. The N‐PASS was used to assess ongoing or acute pain and sedation levels. Head circumference baseline measurement was taken within 7 days before drug administration, or at birth for study patients ≤7 days of age.

#### 2.5.3 Statistical analyses

Planned enrollment for the Exploratory Cohort was six patients. PNA at the time of enrollment, or the time elapsed after birth in days, was calculated as the date of signed inform consent minus the date of birth. PNA in weeks was calculated as PNA in days divided by 7 and rounded up to the nearest week. GA was defined as the number of weeks since the date of the first day of the last menstrual period until birth. CGA was defined as the GA plus the PNA at the date of informed consent. GA was calculated in weeks using PNA in weeks and CGA in weeks with the following formula:

GA=CGA−PNA.

Quantitative variables were summarized using descriptive statistics and categorical variables were summarized using frequencies and percentages. Analysis populations included all patients who received at least one dose of BRV (Safety Set [SS]), and all patients who provided at least one measurable post‐baseline plasma sample (with recorded sampling time) with documented study drug intake times, and who did not have important protocol deviations that potentially could have had a meaningful impact on key PK outcomes (Pharmacokinetic Per‐Protocol Set [PK‐PPS]). Geometric mean (GeoMean) and geometric coefficient of the inter‐individual variation (GeoCV [%]) were calculated for PK parameters. Statistical analyses were carried out using SAS® (Statistical Analysis System) Version 9.4.

## 3 RESULTS

### 3.1 Patients

A total of nine patients were enrolled, of whom three patients were ineligible (responded to initial ASM *n* = 2; elevated liver enzymes *n* = 1); therefore six patients were enrolled into the Exploratory Cohort and treated with at least one dose of BRV. All six patients were included in the SS and the PK‐PPS and completed the evaluation period. Four patients proceeded to the safety follow‐up visit, and two patients completed the BRV extension period and entered the long‐term follow‐up study.

At baseline, patients had a median (range) PNA of 1.5 (1.0, 6.0) days, and a median (range) CGA of 39.0 (36.0, 41.0) weeks (five out of six patients were ≥37 weeks GA) (Table 1). The most common primary cause of seizure was HIE, reported in three patients. All six patients were taking PB at study entry, four were taking MDZ, three were taking PHT and one was taking LEV. Four patients were taking concomitant ASMs during the study, most commonly PB (three patients) and MDZ (three patients).

**TABLE 1:** Baseline demographics, epilepsy characteristics, and ASMs (SS).

	All patients (*N* = 6)
Postnatal age^a^, median (range), days	1.5 (1.0, 6.0)
Corrected gestational age, median (range), weeks	39.0 (36.0, 41.0)
Gestational age^b^, median (range), weeks	38.0 (35.0, 40.0)
Gestational age^b^ < 37 weeks, *n* (%)	1 (16.7)
Gestational age^b^ ≥ 37 weeks, *n* (%)	5 (83.3)
Female, *n* (%)	4 (66.7)
Weight, median (range), g	3110.0 (2550.0, 4300.0)
Length, median (range), cm	52.5 (45.0, 57.0)^c^
Head circumference, median (range), cm	34.5 (31.0, 38.5)
Apgar score (1 min), median (range)	5.5 (0, 10.0)
Apgar score (5 min), median (range)	8.0 (3.0, 10.0)
Suffered from HIE, *n* (%)	3 (50.0)
HIE severity, median (range), Thompson score^d^	14.0 (13.0, 19.0)
Previous and ongoing medical history conditions^e^, *n* (%)	
Any previous and ongoing medical history conditions	4 (66.7)
Medical history conditions in ≥30% of patients, *n* (%)	
Asphyxia	2 (33.3)
Brain edema	2 (33.3)
Cardiovascular insufficiency	2 (33.3)
Hypotonia	2 (33.3)
Respiratory failure	2 (33.3)
Primary cause of seizure^f^, *n* (%)	
HIE	3 (50.0)
Inborn error of metabolism	1 (16.7)
Ischemic stroke	1 (16.7)
Other	1 (16.7)
ASMs taken at study entry^g^, *n* (%)	
At least one ASM	6 (100.0)
Phenobarbital^h^	6 (100.0)
Midazolam^i^	4 (66.7)
Phenytoin^j^	3 (50.0)
Levetiracetam	1 (16.7)
Concomitant ASMs^k^	
At least one ASM	4 (66.7)
Midazolam^i^	3 (50.0)
Phenobarbital^h^	3 (50.0)
Levetiracetam	1 (16.7)
Concomitant hypothermia treatment^l^	3 (50.0)

*Note*: Baseline was defined as the latest assessment before the first dose of IV BRV.

Abbreviations: ASM, antiseizure medication; BRV, brivaracetam; HIE, hypoxic‐ischemic encephalopathy; IV, intravenous; SS, Safety Set.

^a^ The number of days between the date of signed informed consent and the date of birth.

^b^ The number of weeks between corrected gestational age and postnatal age.

^c^ *n* = 5.

^d^ Thompson score was calculated by adding the scores from all nine aspects of the neurological examination of infants with HIE: the total score ranges from 0 to 22 and the kappa coefficient is 0.87 (in normothermic infants, a maximum score > 10 during the first 7 days of life predicts an abnormal outcome with 100% sensitivity and 61% specificity).^31^

^e^ Any previous and ongoing medical conditions that occurred before or at the time of study entry.

^f^ Based on the latest data collected for each patient before study completion/termination.

^g^ ASMs included benzodiazepines and opiates taken by the mother at the time of delivery.

^h^ Phenobarbital included phenobarbital sodium, methylphenobarbital, metharbital, alepsal, phenobarbital, kaneuron, and epanal.

^i^ Benzodiazepine ASMs that could be grouped by bromazepam, alprazolam, cloxazolam, diazepam group, chlordiazepoxide, clonazepam, clobazam, lorazepam, clotiazepam, temazepam, and clorazepate were considered the same ASM at the group level.

^j^ Phenytoin included phenytoin sodium, phenytoin calcium, mephenytoin, zentronal, metetoin, ethotoin, albutoin, hydantal, phelantin, hydantol D, anirrit, dintoinale, fosphenytoin sodium, phenytoin, fosphenytoin, hydantoin derivatives, and hydantoin.

^k^ ASMs taken during administration of BRV. In the event of ambiguity or incomplete data which made it impossible to determine whether a medication was concomitant or not, the mediation was considered concomitant. Combination ASMs were not considered for grouping. Medications were coded using the World Health Organization Drug Dictionary Version September 2017.

^l^ Treatment for hypothermia at any point during the study.

### 3.2 BRV exposure

During the overall study (evaluation and BRV extension period), three patients received one dose of 0.5 mg/kg BRV and three patients received >1 dose. The median (range) duration of exposure to BRV (IV and oral solution) was 1.5 (1.0, 29.0) days (*n* = 6). During the evaluation period, the median (range) duration of exposure to IV BRV was 1.5 (1.0, 3.0) days. For the two patients that entered the BRV extension period, the total median (range) duration of exposure to BRV was 26.5 (0.7) days; this included 12.0 (4.2) days of exposure to IV BRV, and 15.5 (5.0) days of exposure to BRV oral solution.

### 3.3 Pharmacokinetics

Due to the small number of patients enrolled, and since sparse sampling was used in the study, noncompartmental analysis could not be used to analyze BRV data; instead, modeling and simulation were used.

### 3.4 Plasma concentrations and PK parameters of BRV after BRV IV administration

BRV was quantifiable in the plasma at all PK timepoints assessed in patients with evaluable samples. On Day 1, the highest plasma concentration of BRV was observed during the 0.5–1 h time period following IV administration (GeoMean [GeoCV]: 0.53 mg/L [15.40%], *n* = 5) and declined thereafter (2–4 h: 0.50 mg/L [28.20%], *n* = 6; 8–12 h: 0.34 mg/L [13.20%], *n* = 5).

On Day 2, the highest GeoMean (GeoCV) plasma concentration of BRV was observed 0.5–1 h following the dose administered at 24 h (24.5–25.0 h: 0.89 mg/L [17.00%], *n* = 3) and declined thereafter (26–28 h: 0.76 mg/L [18.30%], *n* = 2; 32–36 h: 0.36 mg/L [3.10%], *n* = 3).

**FIGURE 2:**
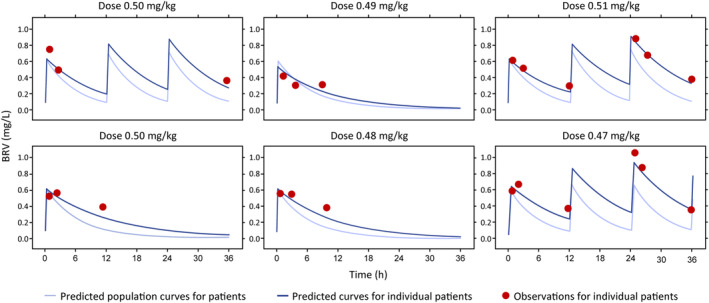
Observed and predicted BRV profiles for individual neonatal patients (PK‐PPS). Individual predicted neonatal curves were calculated using Bayesian feedback. BRV, brivaracetam; PK‐PPS, Pharmacokinetic Per‐Protocol Set.

Individual and population BRV profiles were estimated using Bayesian feedback, and individual PK parameters were calculated (Figure 2). The observed concentrations were consistent with the predicted PK.

GeoMean (GeoCV) values for AUC_(0–12)_ and AUC of BRV were 4.44 h*mg/L (9.10%) and 6.75 h*mg/L (12.80%), respectively (Table 2). The maximum BRV plasma concentration (GeoMean [GeoCV]) was 0.60 mg/L (7.30%), with a median (range) time to maximum plasma concentration of 15 min (15.0, 30.0), and a half‐life (GeoMean [GeoCV]) of 7.65 h (8.30%). GeoMean (GeoCV) values for volume of distribution and clearance were 2.57 L (27.10%) and 0.23 L/h (30.40%), respectively.

**TABLE 2:** Plasma PK parameters of BRV after IV administration (PK‐PPS).

	All patients (*N* = 6)		Individual patient data					
	GeoMean (GeoCV [%])	Median (range)	Patient 1	Patient 2	Patient 3	Patient 4	Patient 5	Patient 6
AUC_(0–12)_ (h*mg/L)	4.44 (9.10)	4.55 (3.71, 4.75)	4.44	4.54	4.75	3.71	4.72	4.55
AUC_(0–infinity)_ (h*mg/L)	6.75 (12.80)	6.98 (5.39, 7.68)	6.36	7.05	7.39	5.39	7.68	6.90
*C*_max_ (mg/L)	0.60 (7.30)	0.62 (0.52, 0.63)	0.63	0.61	0.63	0.52	0.61	0.62
*t*_max_ (min)	16.84 (28.90)	15.00 (15.00, 30.00)	15.00	15.00	30.00	15.00	15.00	15.00
*V*_d_ (L)	2.57 (27.10)	2.52 (1.85, 3.97)	2.18	2.28	1.85	3.97	2.75	2.87
CL (L/h)	0.23 (30.40)	0.22 (0.16, 0.39)	0.22	0.20	0.16	0.39	0.22	0.26
CL (mL/min)	3.88 (30.30)	3.68 (2.71, 6.50)	3.67	3.31	2.71	6.50	3.69	4.35
*t*_½_ (h)	7.65 (8.30)	7.77 (6.87, 8.60)	6.87	7.95	7.90	7.06	8.60	7.63

*Note*: GeoMeans and GeoCVs were only calculated if at least two‐thirds of the parameters were properly determined parameters (ie, non‐calculated and non‐flagged).

Abbreviations: AUC, area under the curve; BRV, brivaracetam; CL, drug clearance; *C*_max_, maximum plasma concentration; GeoCV, geometric coefficient of variation; GeoMean, geometric mean; IV, intravenous; PK‐PPS, Pharmacokinetic Per‐Protocol Set; *t*_½_, elimination half‐life; *t*_max_, time to reach *C*_max_; *V*_d_, volume of distribution.

### 3.5 Plasma concentrations of BRV metabolites after BRV IV administration

GeoMean (GeoCV) plasma concentrations of the BRV acid metabolite increased over the 12‐h dosing interval from 0.02 mg/L (34.10%) (*n* = 5) at 0.5–1 h, to 0.04 mg/L (48.30%) between 2 and 4 h (*n* = 6), and 0.07 mg/L (69.90%) between 8 and 12 h (*n* = 5). GeoMean (GeoCV) plasma concentrations of the BRV hydroxy metabolite were not calculable at 0.5–1 h (*n* = 5), 0.00 mg/L (213.90%) between 2 and 4 h (*n* = 6), and 0.01 mg/L (269.20%) between 8 and 12 h (*n* = 5). GeoMean (GeoCV) plasma concentrations of the BRV hydroxy acid metabolite were not calculable at any timepoint, as concentrations were generally below the lower limit of quantification. The plasma concentrations of the BRV metabolites were all calculable between 24.5 and 36 h (Table S1).

### 3.6 Plasma concentrations of concomitant phenobarbital and phenytoin after BRV IV administration

Between 2 and 4 h after IV administration of BRV, GeoMean (GeoCV) plasma concentrations of PB (*n* = 5) were 42.76 mg/L (36.50%). Plasma concentrations could not be calculated for PHT (*n* = 5), as values for two patients were below the lower limit of quantification.

### 3.7 Tolerability

Three patients experienced four TEAEs, none of which were considered related to BRV (Table 3). No patients discontinued due to TEAEs, and no deaths were reported. One patient (16.70%) had a serious TEAE of apnea that was considered severe in intensity and required mechanical ventilation. This TEAE was considered related to MDZ treatment and did not lead to the patient's discontinuation from the study.

**TABLE 3:** Incidence of TEAEs after IV administration of BRV (SS).

TEAEs, *n* (%)	All patients (*N* = 6)
Any TEAEs	3 (50.0)
Drug‐related TEAEs	0
Serious TEAEs	1 (16.7)
Severe TEAEs	1 (16.7)
Discontinuation due to TEAEs	0
Deaths	0
Individual TEAEs^a^	
Anemia	1 (16.7)
Apnea	1 (16.7)
Dry eye	1 (16.7)
Hyperglycemia	1 (16.7)

Abbreviations: BRV, brivaracetam; IV, intravenous; SS, Safety Set; TEAE, treatment‐emergent adverse event.

^a^ Preferred term (Medical Dictionary for Regulatory Activities 18.1).

Mean and median changes from baseline in important vital signs (including SBP, DBP, heart rate, and respiration rate) were temporary and returned to baseline within 24–48 h. Mean and median changes from baseline to 48 h in laboratory hematology and biochemistry variables were not considered to be clinically meaningful, with values generally within normal ranges. No trends were identified, and no patients showed evidence of potential drug‐induced liver injury.

Median (range) changes in N‐PASS sedation score (*n* = 6) and N‐PASS pain/agitation score (*n* = 5) from baseline to 48 h after BRV administration were 2.0 (−9.0, 5.0), and 0 (−5.0, 2.0), respectively. N‐PASS results were generally within normal ranges; no trends were identified. Median (range) changes in length (*n* = 2), body weight (*n* = 4), and head circumference (*n* = 4) from baseline to safety follow‐up were 0.4 (−1.0, 1.8), 0.9 (0.3, 1.3), and 2.1 (1.7, 3.0), respectively. These changes were generally within normal ranges; no trends were identified. Four patients required mechanical ventilation: three patients were on mechanical ventilation before entering the evaluation period and continued mechanical ventilation during the evaluation period, and one patient initiated and remained on mechanical ventilation for a mean of 1.0 h during the evaluation period.

## 4 DISCUSSION

In this first BRV study in neonates with electroencephalographic seizures, BRV was quantifiable in plasma at all PK timepoints assessed in patients with evaluable samples. Plasma concentrations of BRV rose rapidly, peaking at 15 min and declining thereafter. Plasma concentrations of all BRV metabolites were lower than those of BRV. Plasma concentrations of the BRV acid metabolite were detectable at all assessed timepoints and increased over the 12‐h dosing interval, whereas the BRV hydroxy metabolite, and the BRV hydroxy acid metabolite were lower and/or unquantifiable. Plasma concentrations of concomitant PB between 2 and 4 h after BRV administration (GeoMean 42.76 mg/L) were slightly higher than the therapeutic range of 15–40 mg/L reported in previous neonatal studies.^15^, ^16^, ^17^ Plasma concentrations of PHT were not calculable.

The time course of drug exposure in patients can be described by population PK models, which enable sources of variability in patient exposure to be investigated and alternative dose regimens to be simulated.^18^ A population PK model that described the administration of 2.0 mg/kg b.i.d (maximum of 100 mg b.i.d) BRV oral solution in pediatric patients with epilepsy aged 1 month to 16 years predicted similar plasma concentrations as in adult patients receiving up to 200 mg/day.^14^ Extrapolation of the model to neonates suggested that doses of 2.0–3.0 mg/kg b.i.d might be required to achieve exposure similar to adults receiving the maximum dose of 200 mg b.i.d. A dose of BRV 0.5 mg/kg b.i.d was selected for the current study because this dose offers a wide safety margin (four‐fold) with respect to the highest BRV dose administered and tolerated in older children and adults with epilepsy.^19^ The observed BRV plasma concentrations in neonates were consistent with the PK observed in older children,^14^ and with data from adults receiving a nominal IV dose of 25 mg b.i.d.^20^ In trials of adjunctive BRV, 25 mg b.i.d was shown to be effective in adult patients with focal‐onset seizures.^21^, ^22^ Therefore, a neonatal dose of 0.5 mg/kg b.i.d has potential therapeutic benefits.

Treatment with BRV 0.5 mg/kg b.i.d was well tolerated. Four TEAEs were reported in three patients, and no TEAEs were considered drug related or led to discontinuation. Hematology and clinical chemistry values, physical and neurological examination findings, N‐PASS score, and biometric parameters, were generally within the normal ranges, and no trends were identified. Changes in vital signs (SBP, DBP, heart rate, respiration rate) were temporary, and returned to baseline within 24–48 h.

This study originally planned to recruit a total of 42 patients; however, it was stopped prematurely due to enrollment challenges. The protocol was approved in December 2016 and then amended in October 2019 after the first eligible patient was enrolled, and by May 2021, only six eligible patients had been enrolled. Other studies that have sought to investigate ASMs for the treatment of neonatal seizures have also been terminated because of inadequate enrollment rates.^23^, ^24^

These enrollment challenges reflect the difficulties in conducting interventional clinical studies for neonatal seizures. Studies involving neonates face both ethical and practical challenges. Parents are under immense emotional stress as they adapt to life with a sick newborn, and are unlikely to have an established relationship with the healthcare provider. The decision about whether to provide informed consent to enroll their newborn into a clinical study must be made quickly,^25^ with limited time to understand study information. Factors influencing enrollment in neonatal trials include the parent's perception of the severity of their newborns' illness, trust in medical research,^25^ and the complexity of the consenting process.^26^ In the current study, the exclusion criterion of “seizures responding to previous ASM treatment” may also have limited recruitment.

The International Neonatal Consortium (INC) has developed consensus recommendations with the aim of developing a master protocol to aid in the design of efficient and successful clinical trials.^27^ As per INC consensus recommendations^27^ and the ILAE neonatal classification framework^1^ only neonatal patients with electroencephalographic seizures confirmed by video‐EEG, via local or central EEG reader, were included in the current study. This may have also contributed to the recruitment challenge, given the difficulty for study sites to conduct video‐EEGs. Video‐EEG in neonatal intensive care units is labor intensive, owing to the need for an expert video‐EEG reader to be available on a 24‐h basis to provide rapid expert opinion in real time.^28^, ^29^ Despite these difficulties, video‐EEG is necessary as neonatal seizures are difficult to detect.^30^

### 4.1 Study limitations

Due to the small number of patients that were included in this study, the results and conclusions on safety and tolerability are limited. No efficacy data were collected and no conclusions on efficacy can be made.

## 5 CONCLUSIONS

BRV plasma concentrations in neonates were consistent with predicted data in older children receiving BRV oral solution, and with data from adults receiving a nominal IV dose of 25 mg b.i.d. BRV was generally well‐tolerated, with no drug‐related TEAEs reported.

## AUTHOR CONTRIBUTIONS

All authors made substantial contributions to study conception/design, or acquisition/analysis/interpretation of data; and drafting of the manuscript, or revising it critically for important intellectual content. All authors provided final approval of the manuscript.

## FUNDING INFORMATION

This study was funded by UCB Pharma, which was involved in the design of the study, the collection, analysis, interpretation of data, and manuscript review, and in the decision to publish the manuscript.

## CONFLICT OF INTEREST STATEMENT

R Pressler acts as an Investigator for studies with Johnson & Johnson and UCB Pharma; has received consulting fees and/or honoraria from Eisai, GW Pharmaceuticals, Natus, and UCB Pharma; is supported by the National Institute of Health Research (NIHR) Biomedical Research Centre at Great Ormond Street Hospital, Cambridge Biomedical Research Centre, NIHR, and GOSH Charity. G. Boylan is founder and shareholder in Kephala Ltd and CergenX; and has received consulting fees and/or honoraria from GW Pharmaceuticals, Nihon Kohden, and UCB Pharma. E Dempsey has no conflicts of interest to declare. KA Klotz has received honoraria for lectures and advice from Eisai, GW Pharmaceuticals, Neuraxpharm, and Zogenix. J van den Anker received consulting fees from UCB Pharma for his role on the data monitoring committee for the N01349 study. W Krauwinkel, E Will, D Morita, F Floricel, and JP Elshoff are employees of UCB Pharma.

## ETHICS STATEMENT

We confirm that we have read the Journal's position on issues involved in ethical publication and affirm that this report is consistent with those guidelines.

## PATIENT CONSENT STATEMENT

Parents or legal representatives of patients provided written informed consent before study participation. During the study, continuous consent was utilized via meetings between the Investigator and the parent or legal representatives of the patient to discuss the patient's ongoing care and study participation.

## Supporting information


Table S1:

## Data Availability

Underlying data from this manuscript may be requested by qualified researchers 6 months after product approval in the US and/or Europe, or global development is discontinued, and 18 months after trial completion. Investigators may request access to anonymized individual patient‐level data and redacted trial documents which may include: analysis‐ready datasets, study protocol, annotated case report form, statistical analysis plan, dataset specifications, and clinical study report. Prior to use of the data, proposals need to be approved by an independent review panel at www.Vivli.org and a signed data sharing agreement will need to be executed. All documents are available in English only, for a prespecified time, typically 12 months, on a password protected portal.
