# Supplementary material for: Pharmacokinetics and safety of brivaracetam in neonates with repeated electroencephalographic seizures: A multicenter, open‐label, single‐arm study
Source: Epilepsia Open. 2024 Jan 11;9(2):522–33. doi: 10.1002/epi4.12875 (PMC10984296; doi:10.1002/epi4.12875)
Supplement: Supplementary file 1 — Table S1: [file EPI4-9-522-s001.docx]

# SUPPORTING INFORMATION

# TABLE S1 **Plasma concentrations of BRV metabolites after BRV IV administration following dose on Day 2**

| **GeoMean (GeoCV[%])** | **All patients (*N* = 3)** | **Individual patient data** | | |
| --- | --- | --- | --- | --- |
|  |  | **Patient 1** | **Patient 2** | **Patient 3** |
| BRV acid metabolite  24.5–25 hours | 0.06 mg/L (75.40) | 0.04 mg/L | 0.05 mg/L | 0.14 mg/L |
| BRV acid metabolite  26–28 hours | 0.08 mg/L (86.40)^a^ | - | 0.05 mg/L | 0.14 mg/L |
| BRV acid metabolite  32–36 hours | 0.04 mg/L (101.40) | 0.02 mg/L | 0.04 mg/L | 0.10 mg/ |
| BRV hydroxy metabolite  24.5–25 hours | 0.06 mg/L (88.00) | 0.05 mg/L | 0.04 mg/L | 0.15 mg/L |
| BRV hydroxy metabolite  26–28 hours | 0.08 mg/L (150.90)^a^ | - | 0.04 mg/L | 0.18 mg/L |
| BRV hydroxy metabolite  32–36 hours | 0.07 mg/L (128.90) | 0.04 mg/L | 0.04 mg/L | 0.21 mg/L |
| BRV hydroxy acid metabolite  24.5–25 hours | 0.03 mg/L (162.20) | 0.02 mg/L | 0.02 mg/L | 0.13 mg/L |
| BRV hydroxy acid metabolite  26–28 hours | 0.05 mg/L (224.70)^a^ | - | 0.02 mg/L | 0.13 mg/L |
| BRV hydroxy acid metabolite  32–36 hours | 0.04 mg/L (230.30) | 0.02 mg/L | 0.02 mg/L | 0.18 mg/L |

^a^*n* = 2.

GeoMeans and GeoCVs were only calculated if at least two-thirds of the parameters were properly determined parameters (i.e., non-calculated and non-flagged).

BRV, brivaracetam; GeoCV, geometric coefficient of variation; GeoMean, geometric mean; IV, intravenous; PK PPS, Pharmacokinetic Per-Protocol.
